# Acknowledgment to the Reviewers of *Proteomes* in 2022

**DOI:** 10.3390/proteomes11010004

**Published:** 2023-01-13

**Authors:** Proteomes Editorial Office

**Affiliations:** MDPI AG, St. Alban-Anlage 66, 4052 Basel, Switzerland

High-quality academic publishing is built on rigorous peer review. *Proteomes* was able to uphold its high standards for published papers due to the outstanding efforts of our reviewers. Thanks to the efforts of our reviewers in 2022, the median time to first decision was 21 days and the median time to publication was 56 days. Regardless of whether the articles they examined were ultimately published, the editors would like to express their appreciation and thank the following reviewers for the time and dedication that they have shown *Proteomes*:
Anna Maria SalzanoNagesh PeddadaAnne FasslNathan P. ManesAntonella RosiNobuaki OkumuraAntonio Hernandes Chaves-NetoOrlando CastellanoArmando CaseiroPetro E. PetridesArun Kumar Hanumana Gouda PatilPrabhu PonnandyBhaswati SenguptaPratik JagtapCarlo SantambrogioQuanwei ZhangDaniel RoethRachele TamburinoDavid RamsdenRainer CramerDrashya SharmaRaj KumarElżbieta TrafnyRavi Chand BollineniEvgenia ShishkovaRemigiusz BąchorFederica BonoRenata Del CarratoreFlorence NaillatRobert StryińskiFrancesca LavatelliRui VitorinoFrancesco Di LorenzoRyan SengerFrédéric HalgandSabine LichteneggerHao-ai ShuiSachin VermaHo TsoiSakshi RajoriaHugo CairesSara ReisImre LengyelShariful IslamIsabel Garcia-DorivalShikha JainIstván EgerszegiShu-Hui ChenJeroen KrijgsveldShveta BathlaJiaqi WangSreelakshmi SreenivasamurthyJinan LiSrikanth MandaJody VykoukalSusana B. BravoJohannes JungSyed Azmal AliJonathan D. HumphriesTamas KoszegiJosé Angelito U. HardilloTao LinKa YangTatu RojalinKavindra Kumar KesariTiantian JiangLasse LindahlTimothy HamerlyLeo Kei IwaiTomasz PowrózekLeo VeenmanTrayambak BasakLucas DelmonicoXaveer Van OstadeLydie Nadal-DesbaratsXiming YuanMałgorzata GrzankaXiucong BaoMaria Cristina Baracat-PereiraYi-Ting WangMariusz NiemczykYoshitaka FukuzawaMariusz Tomasz DziadasYuan GaoMartina SamiotakiYu-Hsiang KuanMasaru MiyagiYujia WangMikhail Ivanovich VoevodaZeeshan HamidMukesh Kumar



